# Synchronizing beta cells in the pancreas

**DOI:** 10.7554/eLife.95103

**Published:** 2024-01-25

**Authors:** Bradford E Peercy, David J Hodson

**Affiliations:** 1 Department of Mathematics and Statistics, University of Maryland Baltimore County (UMBC) Baltimore United States; 2 Oxford Centre for Diabetes, Endocrinology and Metabolism (OCDEM), NIHR Oxford Biomedical Research Centre, Churchill Hospital, Radcliffe Department of Medicine, University of Oxford Oxford United Kingdom

**Keywords:** islet, network theory, coordinated oscillations, beta-cell oscillations, insulin release, beta-cell, heterogeneity, Mouse

## Abstract

The secretion of insulin from the pancreas relies on both gap junctions and subpopulations of beta cells with specific intrinsic properties.

**Related research article** Briggs JK, Gresch A, Marinelli I, Dwulet JM, Albers DJ, Kravets V, Benninger RKP. 2023. β-cell intrinsic dynamics rather than gap junction structure dictates subpopulations in the islet functional network. *eLife* **12**:e83147. doi: 10.7554/eLife.83147.

The amount of glucose in the blood is controlled by the hormone insulin, which is released by the pancreas when glucose levels get too high. The hormone is released from beta cells that are organized into spheroid structures within the pancreas known as islets of Langerhans. Like muscle cells in the heart, beta cells are electrically coupled together by gap junctions, and this coupling enables the cells within the islet to coordinate or synchronize their behavior and release insulin in a pulsatile manner.

Gap junctions are thought to be critical for controlling the dynamics of the islets and, hence, insulin secretion. Indeed, mice lacking gap junctions are unable to release insulin in pulses (Head et al., 2012). Gap junction (or electrical) coupling between beta cells has also been shown to weaken with age as insulin secretion declines and individuals become more susceptible to type 2 diabetes (Westacott et al., 2017a).

Recent studies have shown that beta cells can be separated into subpopulations based on their genetic makeup, the proteins they make, and how they behave (Benninger and Hodson, 2018). Some of these subgroups have a greater influence over islet dynamics than others (Stožer et al., 2013; Johnston et al., 2016; Salem et al., 2019; Westacott et al., 2017b; Nasteska et al., 2021). When these cells are disrupted – either by optogenetics or gene overexpression – islet function and insulin secretion decline, reminiscent of what occurs during aging and type 2 diabetes.

These subpopulations of beta cells are not physically connected and instead rely on their intrinsic properties to influence islet dynamics (Johnston et al., 2016; Westacott et al., 2017b; Nasteska et al., 2021). However, the cells in these subpopulations are too few in number to influence electrical coupling by gap junctions (Peercy and Sherman, 2022). Additionally, gap junctions alone cannot explain the activity patterns of the subpopulations identified, or their influence over islet function. So how do these two mechanisms work together to control blood glucose levels? Now, in eLife, Richard Benninger and co-workers – including Jennifer Briggs as first author – report new findings that shine some light on the relationship between beta cell subpopulations and gap junctions (Briggs et al., 2023).

The researchers (who are based at the University of Colorado Anschutz Medical Campus and the University of Birmingham) found that the enzyme glucokinase – which senses changes in blood glucose levels – displayed elevated levels of activity in a subpopulation of beta cells. This resulted in heightened metabolism due to glucokinase breaking down more molecules of glucose to generate the high levels of ATP (usable energy) versus ADP (used energy) required for insulin release, reflecting previous findings (Johnston et al., 2016; Westacott et al., 2017b).

Notably, beta cells were more likely to synchronize their response to glucose if their metabolic activity was similar; moreover, changing these intrinsic properties led to a loss of the beta cell subpopulation. Reducing gap junction coupling also did not stop the beta cells within the islet from synchronizing their activity. It did, however, make them much weaker at transmitting electrical signals across the islet.

It has long been thought that gap junctions are the major driver of synchronized beta cell activity, and that their disruption during diabetes leads to impaired insulin secretion. However, the findings of Briggs et al. suggest that gap junctions are just one piece of the jigsaw, and that cells with similar intrinsic properties – such as metabolic actvity – also drive islet dynamics (Figure 1).

**Figure 1.**
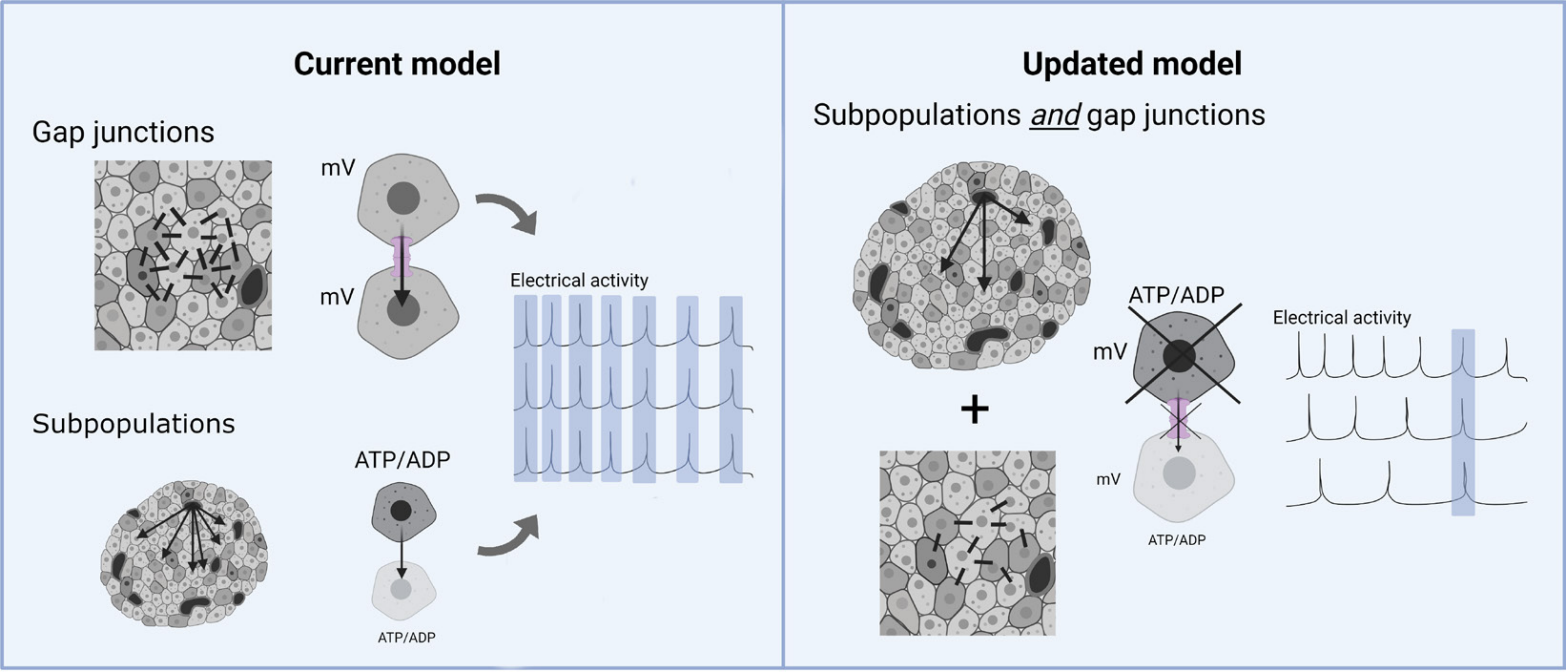
The role of gap junctions and beta cell subpopulations in insulin release. Within the pancreas, insulin-secreting beta cells are arranged into islets, and are physically coupled together by gap junctions (dark gray rectangles/lines). In the current model (left), gap junctions (pink) spread electrical currents (mV) between beta cells within the islet, resulting in the population displaying similar oscillations of electrical activity (right hand graph, matching waves highlighted in blue) and synchronizing their insulin release. Within the islet are also subpopulations of beta cells with specific intrinsic properties (shaded in dark grey; lower panel), such as higher levels of metabolic activity or producing more usable energy (ATP) than used energy (ADP). These beta cell subpopulations also contribute to coordinated beta cell activity, but how exactly was largely unknown. In the updated model proposed by Briggs et al. (right), the beta subpopulations *and* gap junctions work together to control islet dynamics. The increased metabolism of the beta subpopulations makes it easier for gap junctions to spread electrical currents across the islet. Disrupting either of these mechanisms (represented by an X symbol) makes it harder for beta cells within the islet to fully synchronize their electrical activity (left hand graph), leading to a decline in insulin secretion. mV = membrane potential.

Image credit: Figure created with BioRender.com.

So which mechanism fails first during diabetes: gap junctions or intrinsic cellular properties? Small decreases in the number of gap junctions and their associated electrical signalling, which occurs during diabetes, would make it much harder for beta cells within a subpopulation to synchronize. On the other hand, small changes in intrinsic cellular properties might render gap junction synchronization much less effective. Complicating matters further, loss of gap junction coupling likely influences the intrinsic properties of beta cells and vice versa. Therefore, the disrupted islet dynamics and impaired insulin release observed in patients with diabetes is probably due in part to both mechanisms failing simultaneously.

The study by Briggs et al. shows that no single mechanism drives synchronized beta cell activity: rather, subpopulations and gap junctions come together to shape islet behaviour. Further computational modelling could help tease out – or even predict – how the critical relationship between beta cell subpopulations and gap junctions influences insulin release. Further experimental work is also warranted to understand how the interplay between beta cell subpopulations and gap junctions is altered during diabetes.
